# Exploration of Effective Time-Velocity Distribution for Doppler-Radar-Based Personal Gait Identification Using Deep Learning

**DOI:** 10.3390/s23020604

**Published:** 2023-01-05

**Authors:** Keitaro Shioiri, Kenshi Saho

**Affiliations:** Department of Intelligent Robotics, Toyama Prefectural University, Imizu 939-0398, Japan

**Keywords:** biometrics, Doppler radar, gait recognition, person identification, deep learning, time-velocity distribution

## Abstract

Personal identification based on radar gait measurement is an important application of biometric technology because it enables remote and continuous identification of people, irrespective of the lighting conditions and subjects’ outfits. This study explores an effective time-velocity distribution and its relevant parameters for Doppler-radar-based personal gait identification using deep learning. Most conventional studies on radar-based gait identification used a short-time Fourier transform (STFT), which is a general method to obtain time-velocity distribution for motion recognition using Doppler radar. However, the length of the window function that controls the time and velocity resolutions of the time-velocity image was empirically selected, and several other methods for calculating high-resolution time-velocity distributions were not considered. In this study, we compared four types of representative time-velocity distributions calculated from the Doppler-radar-received signals: STFT, wavelet transform, Wigner–Ville distribution, and smoothed pseudo-Wigner–Ville distribution. In addition, the identification accuracies of various parameter settings were also investigated. We observed that the optimally tuned STFT outperformed other high-resolution distributions, and a short length of the window function in the STFT process led to a reasonable accuracy; the best identification accuracy was 99% for the identification of twenty-five test subjects. These results indicate that STFT is the optimal time-velocity distribution for gait-based personal identification using the Doppler radar, although the time and velocity resolutions of the other methods were better than those of the STFT.

## 1. Introduction

In recent times, biometric technology is extensively used in surveillance and monitoring systems, with the most common application being that of facial recognition via camera for authentication purposes [1,2]. In addition, smart speakers have had a recent surge in popularity and are also used for personal identification [3]. However, such techniques have serious privacy concerns. To overcome these privacy issues, personal identification techniques using various biometric information, such as fingerprints, irises, veins, brain waves, and heartbeat characteristics, have been investigated [4,5]. However, the acquisition of such biometric information often requires physical contact with the user and/or a complex setup. Therefore, personal identification based on unconstrained daily activities is an important research topic. In particular, gait-based personal identification has recently been studied, as walking is a representative daily activity that can be easily measured and applied for continuous identification [6,7].

The use of cameras herein is primarily for personal identification using gait information [8,9]; however, there are privacy issues similar to facial recognition, with their accuracy depending on the lighting conditions and a subject’s outfit. Methods using depth sensors have also been studied [10,11]; however, their accuracy depends on the lighting conditions, similar to those in the case of cameras. Moreover, measurement of wide areas is relatively difficult. Another approach is the use of accelerometers, including those used in wearable devices and smartphones [12,13]. However, in this case, the user is required to carry or wear sensors.

Doppler radar is a promising prospect for solving the aforementioned problems with other sensors [14,15]. Doppler radar can remotely measure the time variation in the velocities during human movements based on frequency transitions caused by the Doppler effect. During Doppler radar motion measurement, the participants are not required to wear sensor devices, and there are no restrictions on the participants’ outfits. Gait-based personal identification using the micro-Doppler radar and applying deep learning to time-velocity distribution, which was calculated as the short-time Fourier transform (STFT) of the radar-received signals, has yielded high accuracy [16,17,18,19,20,21,22,23]. It has been reported that the identification of two persons has an accuracy of 99% [16], and identification of 20 persons has an accuracy of 97% [17]. Furthermore, several person identification methods have been recently developed which are suitable for various types of realistic scenarios such as a multi-person scenario [18], use of a relatively small amount of training data [19,20], and scenarios which assume people walking in arbitrary directions [21].

However, most conventional studies on Doppler-radar-based personal identification do not consider the relationships between gait identification accuracy and time-velocity distributions and their resolutions. For example, there are multiple methods for calculating the time-velocity distributions other than STFT, such as wavelet transform (WT) [24], Wigner–Ville distribution (WVD) [25], and smoothed pseudo-WVD (SPWVD) [26]. These time-frequency analysis techniques have been recently used for various signal classification problems based on deep learning techniques, such as electrocardiogram techniques [27,28,29]. Thus, even for the Doppler radar techniques, several researchers have efficiently used such deep-learning- and time-frequency (time-velocity)-based methods for human motion classification problems [30,31,32]. However, methods other than STFT have rarely been applied to gait-based personal identification using Doppler radars. Although Dong et al. [33] presented the effects of time-velocity distribution on gait identification, no significant differences between the STFT and WVD-based methods were observed because the numbers of participants and test trials were limited, and their various parameter settings, which control the time-velocity resolution, were not considered. Thus, an efficient time-velocity distribution and parameter setting have not been established for Doppler-radar-based personal gait identification.

In this study, we explore efficient time-velocity distributions for personal gait identification using Doppler radar and deep learning by comparing the identification accuracy of various time-velocity distributions involving STFT, WT, WVD, SPWVD, and their various parameter settings. The contributions of this study are as follows.

- For gait-based person identification using deep learning and Doppler radar, tuning of micro-Doppler signatures is considered, and the appropriate settings are revealed.
- A comparison of the person identification accuracies of various time-velocity distributions showing that the conventionally used STFT spectrograms achieved the best accuracy.
- Twenty-five test subjects were successfully identified with an accuracy of approximately 99%.

## 2. Radar Gait Measurement and Person Identification Procedure

**Figure 1:**
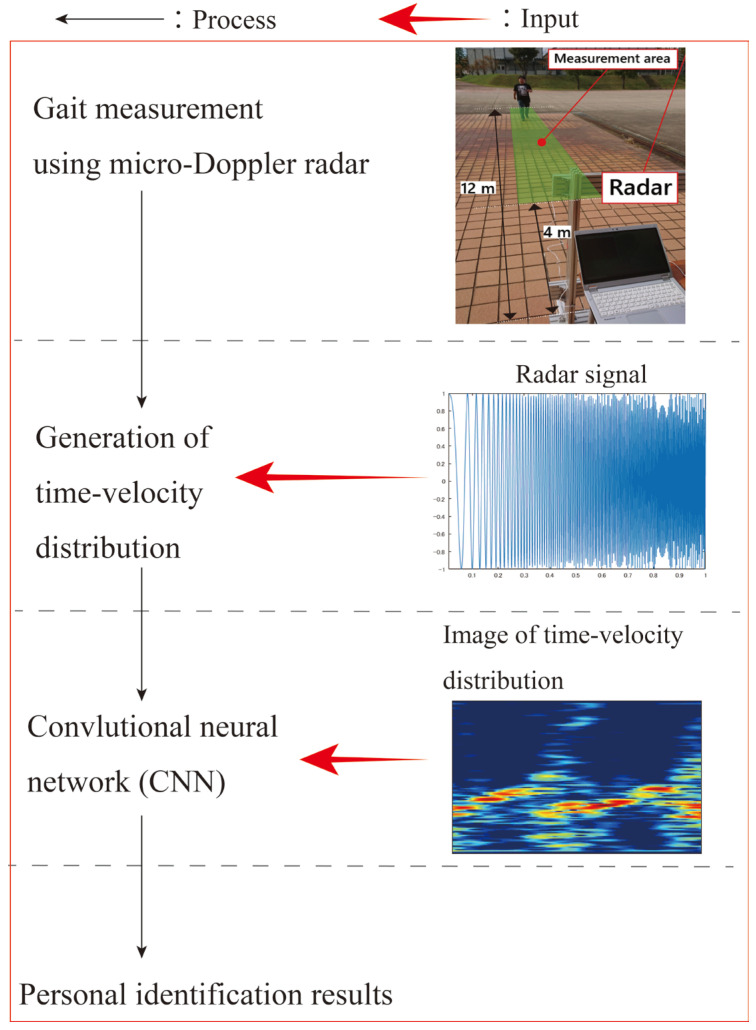
Outline of the procedure used in this study.

Figure 1 outlines the procedure of the Doppler radar experiments and person identification. Gait measurements were performed to generate the dataset. We used a monostatic continuous-wave 24 GHz micro-Doppler radar (ILT Office Inc., Toyama, Japan, BSS-110) installed at a height of 1.0 m. The −3 dB beamwidth in the V- and H-planes of the antenna were ±14° and ±35°, respectively. The received demodulated radar signals were obtained at a sampling frequency of 600 Hz. The study participants comprised 25 healthy adults (mean age: 22.5 years; 22 men and 3 women) who wore their own shoes (none of the participants wore shoes that led to relatively difficult walking). The participants were instructed to walk toward the radar along a straight walkway at a self-selected comfortable pace.

We collected the received signals corresponding to the participants who walked in the range of 4–12 m because they performed steady-state walking, and the radar could measure the entire body in this range. We collected the data for 150 gait cycles for each participant for several days. The received signal of each gait cycle was used as one dataset for personal identification, and a total of 150 × 25 = 3750 data points were collected. The length of each input data point is one gait cycle. We extracted the data corresponding to each gait cycle from the collected data of steady-state walking using the method presented in [34]. Therefore, the length of each data point was approximately 1 s, which is referred to as the general value of the walking cycle of human gait; however, the gait cycle of each data point was different.

We then generated images of the time-velocity distribution calculated using the received signals. The focus of this study is to clarify the type and parameters of the time-velocity distribution that achieves accurate identification. The details involving the generation method for various types of time-velocity distributions and their parameters are explained in Section 3. The identification accuracies for the various types and settings of the generated time-velocity images are compared in Section 4.

We investigated the identification accuracy of the 25 participants using the generated time-velocity distribution and deep learning. A convolutional neural network (CNN) was used as the deep learning method, similar to previous studies on Doppler-radar-based personal identification. The time-velocity distributions converted to RGB-colored PNG images of size 224 × 224 were used as the input data for the CNN. Because we focused on the exploration of the effective time-velocity distribution, the CNN structure used in this study was fixed to ResNet-18 [35], which is an effective network for various conventional radar-based motion and person identification systems [23,36]. The basic structure of ResNet-18 was used with the same empirically tuned parameter settings as in our previous study [14]. Global average pooling was performed, and a fully connected layer was then employed using stochastic gradient descent with momentum optimization. The loss function was a cross-entropy function. The hyperparameters were optimized for all experiments for their fair comparison. For example, for the STFT spectrogram, a mini-batch size of 64. The learning rate was 0.01 and was decreased by multiplying it times 0.7 every 10 epochs. These hyperparameters were empirically optimized.

## 3. Generation of Various Time-Velocity Distribution Images

This section describes the methods for generating the time-velocity distributions: STFT, WT, WVD, and SPWVD. The procedures to calculate various time-velocity distributions and their examples and features are presented herein.

### 3.1. STFT Spectrogram

**Figure 2:**
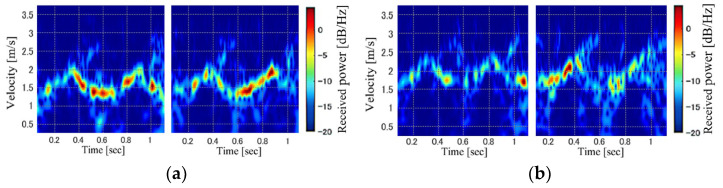

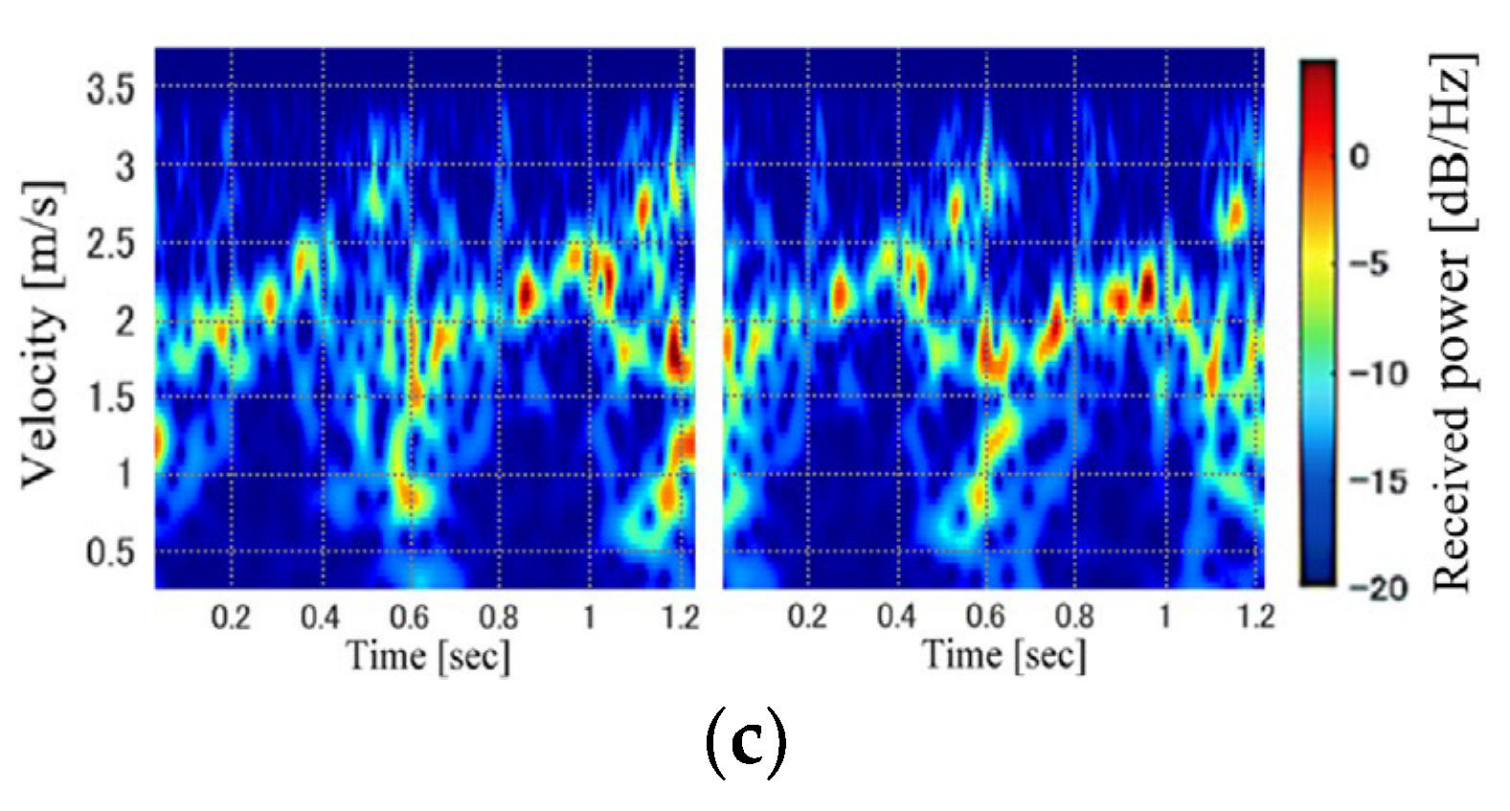
Examples of STFT spectrogram images. Each panel corresponds to a different participant (subfigures (**a**–**c**) show the spectrogram images of participants (**a**), (**b**), and (**c**), respectively).

This section describes the method for generating the time-velocity distributions. This subsection presents the most commonly used STFT methods. The STFT of the radar-received signal *s*(*t*) (t: time) is expressed as [25]:

(1) $$S\left(t,\;\omega_{d}\right)=\int s\left(\tau \right)w\left(\tau -t\right)e^{-j\omega_{d}\tau }d\tau ,\;$$

where *w*(*t*) is a window function, and this study used a general Hamming window function with the length of *W*_L_ (note that we confirmed that the accuracies of the gait identifications presented in this paper are only slightly changed for other representative window functions involving Hann and Blackman window functions). The radial velocity *v*_d_ can be calculated using the Doppler angular velocity *ω*_d_ as:

(2) $$v_{d}=\frac{c}{4\pi f_{0}}\omega_{d},\;$$

where *f*_0_ is the transmitting frequency (24 GHz), and *c* is the speed of light. For micro-Doppler-radar-based personal identification, the magnitude squared of the STFT, which is referred to as a spectrogram, is often used. Using Equation (2), the STFT spectrogram of the received signal |*S*(*t*, *v*_d_)|^2^ is calculated, and its images are input to a machine-learning algorithm, such as a CNN. Figure 2 shows examples of the spectrogram images with the window length *W*_L_ of 128 samples for steady-state gaits of three participants. The similar images for the same participants and different tendencies depending on the participants could be confirmed.

**Figure 3:**
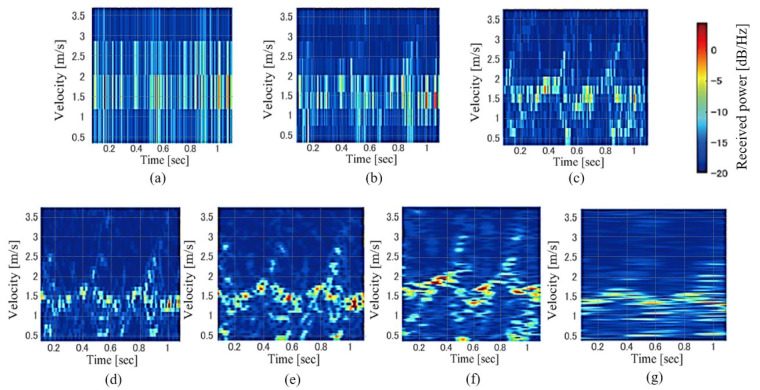
Examples of STFT spectrogram images for various window lengths. *W*_L_ = (**a**) 4, (**b**) 8, (**c**) 16, (**d**) 32, (**e**) 64, (**f**) 128, and (**g**) 256 samples.

Although most studies on micro-Doppler-radar-based personal identification have used STFT spectrogram images, the effects of their resolution, which is determined by the window length *W*_L_, have not been investigated. Figure 3 illustrates examples of the spectrograms of gait for various *W*_L_ calculated using the same data with an overlap length of *W*_L_−1. This figure clearly shows that the velocity (frequency) resolution of the spectrogram improved when a large *W*_L_ was set. However, a larger *W*_L_ results in a lower time resolution. Thus, the expressed information on gait in the spectrogram depends on *W*_L_, and we investigated the relationship between *W*_L_ and personal identification accuracy.

### 3.2. WT Scalogram

WT is another popular time-frequency analysis method owing to its resolution flexibility. The WT of *s*(*t*) is expressed as [32]:

(3) $$\mathrm{WT}\left(a,\;u\right)=\int s\left(\tau \right)\frac{1}{\sqrt{a}}\psi^{*}\left(\frac{\tau -u}{a}\right)d\tau ,$$

where * indicates a complex conjugate, *a* is the scale factor (corresponding to the velocity), *u* is the shift factor, and *ψ*(*t*) is the wavelet function. We used a Morse wavelet [37] as the wavelet function because it can easily control both the time and frequency resolutions, which are expressed as follows:

(4) $$\psi \left(t\right)=\frac{1}{2\pi }\int 2{\left(e\gamma /b\right)}^{b/\gamma }\omega^{b}e^{-\omega^{\gamma }}e^{j\omega t}d\omega ,\;$$

where *b* and *γ* are the parameters that control the time and frequency resolutions, respectively. For personal identification, we used images having the magnitude squared of the WT, called a scalogram.

**Figure 4:**
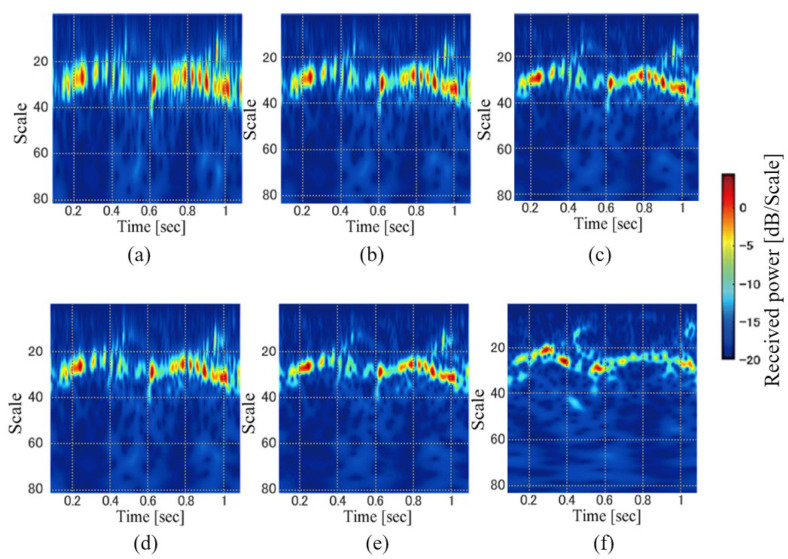
Examples of WT scalogram images. (*b*, *γ*) = (**a**) (8, 3), (**b**) (16, 3), (**c**) (32, 3), (**d**) (8, 8), (**e**) (16, 8), and (**f**) (32, 8).

Figure 4 shows examples of the scalograms for various settings of *b* and *γ*. In contrast to the STFT, the time and scale (velocity) resolutions are not fixed in the scalogram and are controlled by these parameters. We investigated the personal identification accuracy for various parameter settings and compared the results of the WT scalogram with those of the STFT spectrogram.

### 3.3. WVD

WVD is a high-resolution time-frequency analysis method without a trade-off between the time and frequency resolutions that exist in the STFT and WT. The WVD of *s*(*t*) is expressed as [25]:

(5) $$\mathrm{WVD}\left(t,\;\omega_{d}\right)=\int s\left(t+\frac{\tau }{2}\right)s^{*}\left(t-\frac{\tau }{2}\right)e^{-j\omega_{d}\tau }d\tau .\;$$

**Figure 5:**
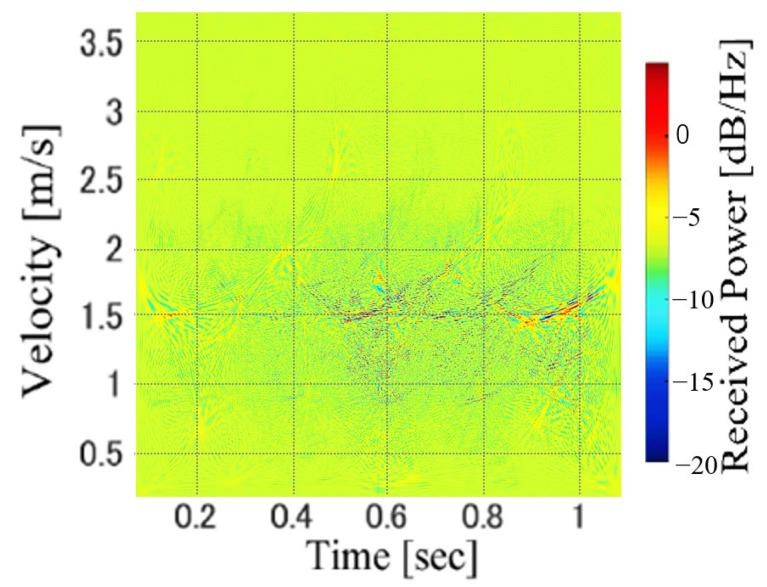
An example of WVD image.

The WVD can determine the frequency spectrum for each time bin, and the time and frequency resolutions are equivalent to the physical limitations determined by the sampling frequency. However, owing to the interference of the multifrequency components, the WVD includes many cross-terms. Figure 5 shows an example of the WVD of the Doppler-radar-received signal of the gait. Compared with the STFT spectrograms shown in Figure 3, the velocity components of the walking motion are not clearly confirmed because of the many cross-terms. Thus, WVD has not been widely used for Doppler radar-based motion recognition. However, we can hypothesize that these cross-terms can be considered as the features of individuals in the personal identification problem. Thus, we investigated the accuracy of personal gait identification using WVD images.

### 3.4. SPWVD

The SPWVD is a smoothed WVD for removing the cross-terms in the WVD; it is expressed as follows [25,26]:

(6) $$\mathrm{SPWVD}\left(t,\;\omega_{d}\right)=\iint \Phi \left(t-t^{\prime },\;\omega_{d}-\omega_{d}^{\prime }\right)\mathrm{WVD}\left(t,\;\omega_{d}\right)dt^{\prime }d\omega_{d}^{\prime },\;\;\;$$

where Φ(*t*, *ω*_d_) is the smoothing function. In this study, we used the two-dimensional Gaussian function for Φ(*t*, *ω*_d_), which is expressed as:

(7) $$\Phi \left(t,\;\omega_{d}\right)=\exp\left(-\frac{t^{2}}{\alpha^{2}}-\frac{\omega_{d}{}^{2}}{\beta^{2}}\right)$$

where *α* and *β* are the parameters that control the resolution in terms of time and velocity, respectively.

**Figure 6:**
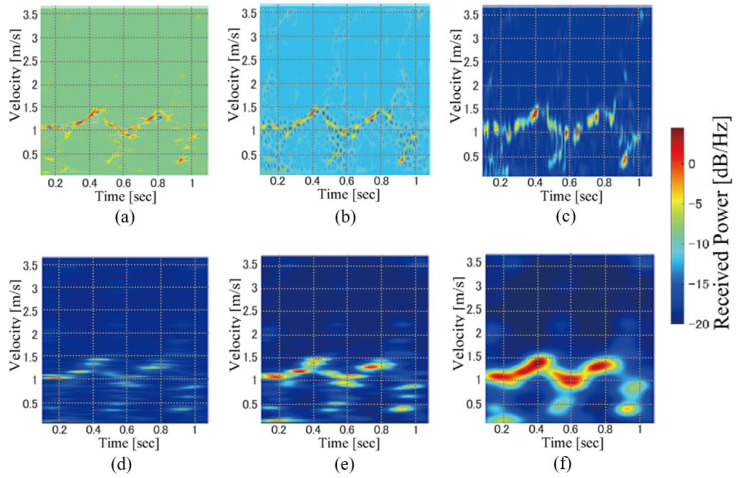
Examples of SPWVD images. (*α*, *β*) = (**a**) (4, 4)*,* (**b**) (4, 16), (**c**) (4, 64), (**d**) (64, 4), (**e**) (64, 16), and (**f**) (64, 64).

Figure 6 shows examples of the SPWVDs for various values of *α* and *β.* When *α* and *β* are set to smaller values, a high-resolution time-velocity distribution is obtained. However, several cross-terms remain with extremely small values. In contrast, larger values of these parameters sufficiently remove the cross-terms, although the resolutions worsen. As shown in Figure 6, SPWVDs with larger parameters tend to produce results that are similar to the spectrograms shown in Figure 3. SPWVDs with smaller parameters are high-resolution time-velocity distributions compared to the STFT and WT with smaller cross-terms compared to the WVD.

As presented in this section, various time-velocity distributions with different parameter settings generate the input images with different resolutions. In the next section, we evaluate the personal gait identification accuracy for the aforementioned time-velocity distribution types and resolution of the input time-velocity images.

## 4. Evaluation and Discussion

### 4.1. Evaluation Method

We evaluated and compared the accuracy of the gait identification of the participants using images of various time-velocity distributions with various parameter settings, as described in the previous section. We also investigated the identification accuracy according to the number of participants *N*; cases with *N* = 5, 15, and 25 participants were considered. When we investigated the identification of five or fifteen participants, the participants were randomly selected from the twenty-five participants. For accuracy evaluation, we performed a hold-out validation. In each case, the CNN was trained using 70% of the data (105 data points per participant), and the remaining 30% of the data were used as test data. Thus, the sizes of training data for *N* = 5, 15, and 25 were 525, 1575, and 2625 images; those of test data for *N* = 5, 15, and 25 were 225, 675, and 1125 images, respectively. The number of training and test data was the same for all time-velocity distributions. Subsequently, ten trials of hold-out validations were conducted by varying the training/test data split. The training and test data were randomly selected from the generated dataset. Notably, the length of each data point is one gait cycle. The mean and standard deviation of the classification accuracy across all trials were calculated.

### 4.2. Results for STFT

Table 1 summarizes the results for the personal gait identification using the STFT spectrogram images for various window lengths *W*_L_ and number of participants *N*. All the *N* and *W*_L_ values of the 32 samples (53.3 ms) achieved the best accuracy of approximately 99%. We achieved accurate gait identification of 99.1% in the identification of 25 participants. The results revealed that the identification accuracy worsens when either the time or frequency resolution is relatively low.

**Figure 7:**
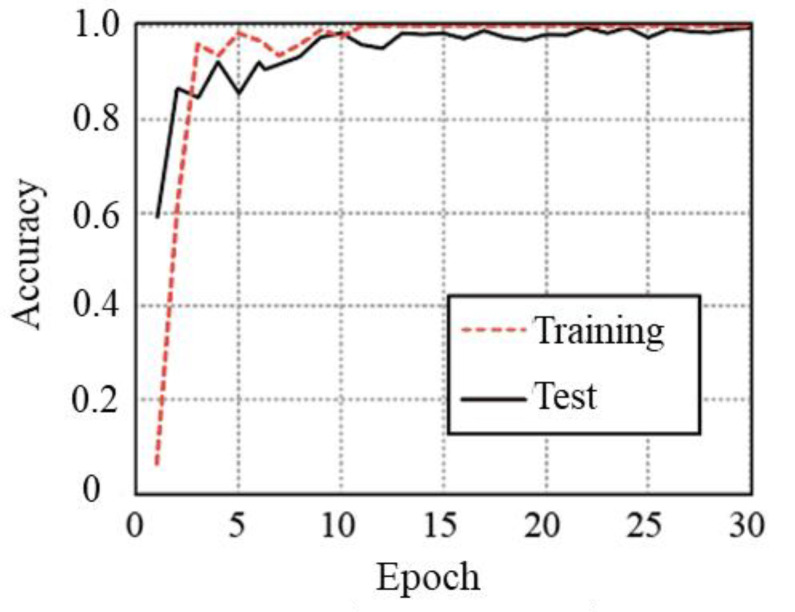
Example of convergence curves for STFT with *N* = 25 and *W*_L_ = 32 samples.

Figure 7 shows an example of the convergence curves obtained for the results of *N* = 25 and *W*_L_ = 32 samples. As shown in these curves, the accuracy is converged with small epochs without overfitting. We confirmed the similar tendencies of the conventional curves for all cases. Thus, there are no requirements for data augmentation for the accurate personal gait identification based on our experimental data.

### 4.3. Results for WT

**Figure 8:**
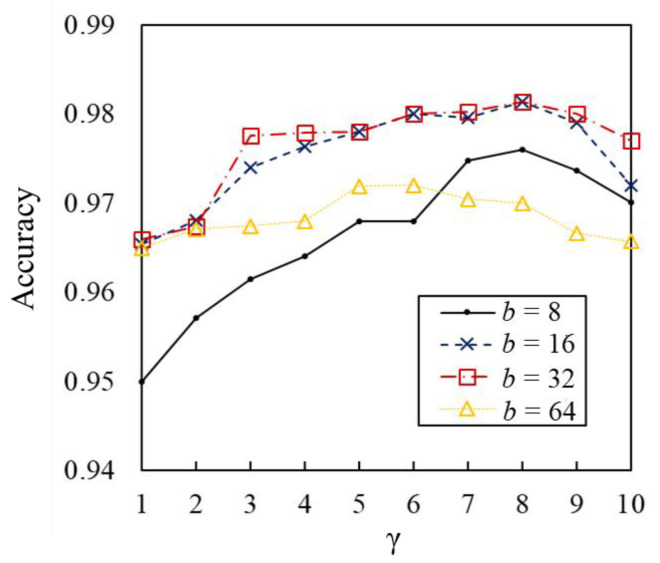
Identification accuracies using WT for various parameters (*N* = 25).

We then investigated the accuracy of the WT scalograms that have flexibility in terms of time and velocity resolution settings. Table 2 lists the results of the WT scalograms for various *b* and *γ* values. For all N values, the identification accuracies for (*b*, *γ*) = (8, 32) achieved the highest accuracy of approximately 98%. Relatively larger values of *b* and *γ* indicate that the resolutions in time and scale are the same. These results indicate that the identification accuracy is low when the time or scale resolution is high. Figure 8 shows the results for WT with various parameter settings for *N* = 25. As shown in this figure, larger values of *b* were ineffective compared with the results for *b* = 16 and 32. Furthermore, larger *γ* values slightly deteriorate the identification accuracy for various *b* values. These results indicate that there are appropriate resolutions of the WT scalograms for the gait-based person identification, similar to the STFT spectrograms.

### 4.4. Results for WVD and SPWVD

**Figure 9:**
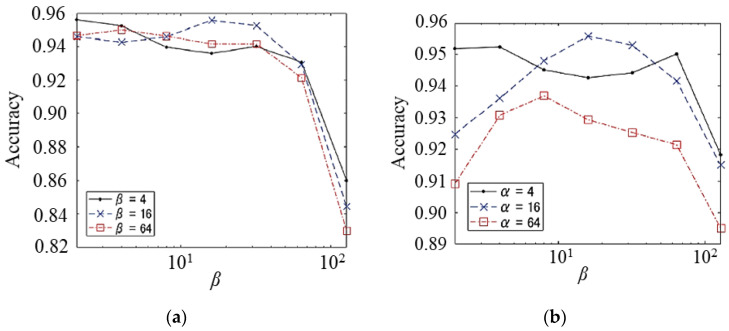
Identification accuracies using SPWVD for various parameters (*N* = 25). Relationships between accuracy and (**a**) *α*, (**b**) *β*.

Subsequently, we examined the effectiveness of the higher-resolution time-velocity distributions of WVD and SPWVD. Table 3 shows the representative results for the WVD and SPWVD. For all *N* values, the SPWVD achieved better accuracy than the WVD with appropriate parameter settings. These results indicate that the suppression of cross-terms was effective for gait identification, despite the reduction in the time-frequency distribution resolution due to the smoothing process in the SPWVD. Figure 9 shows the results for the SPWVD with various parameter settings for *N* = 25. We observed that there exists an optimal setting for both *α* and *β*. However, the optimal accuracy did not exceed 96%, which is worse than the results for STFT and WT.

### 4.5. Overall Comparison and Discussion

The above results include various novel findings for the radar-based personal gait identification method with respect to the efficient time-velocity distribution images and their resolutions. Thus, we compared the results obtained using the various time-velocity distributions to clarify the findings and contributions of this study. Note that this study dealt with the personal identification based on only the natural human walking, and the properties of the signals of all participants and data are similar. Therefore, the discussion based on the time-velocity resolution (e.g., window length of the STFT) is reasonable.

**Figure 10:**
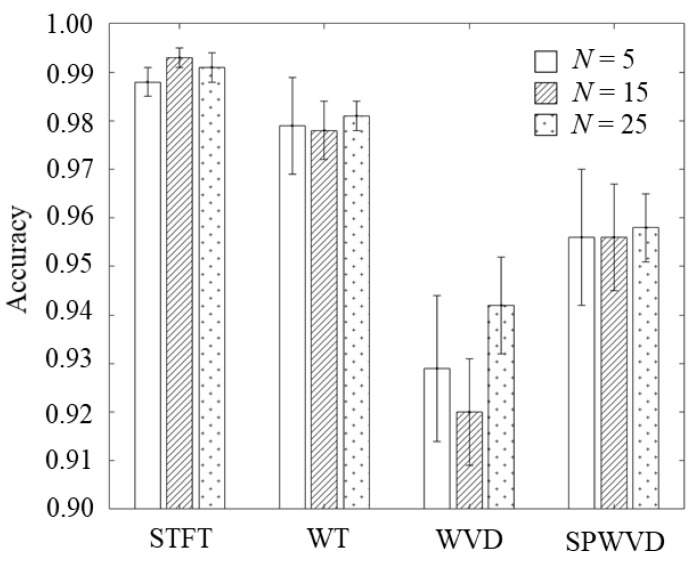
Comparison of identification accuracies using various types of time-velocity distributions.

Initially, we compared our results with those described in the previous subsections. Figure 10 shows the mean and standard deviation of the identification accuracies for the optimal settings of all methods. Although all methods achieved accurate identification of over 92%, the STFT method achieved significantly better results for all *N*. These results indicate that the various time-velocity distributions, which can flexibly control the resolutions and achieve high resolutions, were effective to a certain extent. However, conventionally used STFT has been revealed to be a more effective generation method for time-velocity distribution images for personal gait identification.

We now discuss the reasons for the results that explain the differences in accuracy caused by the time-velocity distribution. A comparison of WVD and SPWVD indicated that the high-resolution WVD was less accurate than the SPWVD. These results imply that the rich information included in the cross-terms of the WVD was ineffective for personal identification. As indicated in the WVD of Figure 5, we can confirm the larger powers of the background noise caused by several cross-terms compared with the case of the SPWVD as shown in Figure 6. Such higher background noise might affect the feature extraction. For the SPWVDs in Figure 6, although the powers of the background noise were larger for smaller α and β, the components corresponding to the body and legs in the gait were clearly confirmed compared to that in the case of WVD. Thus, the background noise caused by the cross-terms of the WVD ineffectively affected gait feature extraction, and SPWVD achieved better identification accuracy by effectively suppressing the cross-terms.

The ineffectiveness of the cross-terms can be appraised from the comparison between SPWVD and STFT, and we can confirm from the results that the SPWVD was ineffective even when the resolution of the time-velocity distribution was similar to that of the STFT method. These results imply that the cross-terms in the WVD and SPWVD images were not effectively used for feature extraction of the individuals. Furthermore, the cross-term suppression effects in SPWVD images are unstable and may lead to deterioration of identification accuracy.

The comparison between STFT and WT also slightly, but significantly, indicates better effectiveness of the STFT method despite their similar resolutions (both STFT and WT have a trade-off between time and velocity resolutions), and the flexibility in the resolution setting of the WT is better than that of the STFT. These results imply that the STFT spectrogram includes sufficient and clear information on individual gait features, and its flexible tuning via WT does not result in additional gait features. One reason why the STFT can grasp sufficient gait features is that the gait identification based on steady-state gait was considered in this study; transient state of acceleration and deceleration in walking is not assumed. Although the WT scalogram is suitable for the acquisition of transient features based on scaling, this merit might not translate toward the extraction of features in steady-state walking. It is conjectured that extremely detailed information, which is ineffective for gait identification based on steady-state walking, was extracted via the WT scalogram owing to its flexibility in time and velocity resolutions. In fact, the identification accuracy of the STFT spectrogram with *W*_L_ of 32 samples was better than that of the WT scalogram with (*b*, *γ*) = (32, 3) despite their similar resolutions. Thus, the STFT spectrogram is an appropriate method for gait-based person identification using a Doppler radar.

We discuss the factors that influence gait-based personal identification using the Doppler radar. As shown in Figure 2, it can be seen that participants differ in terms of received power, trunk velocity, and leg kinetic velocity. While these are all factors that identify personal gaits, the most important factor would be the leg velocity data. This is because the WVD cross-terms mask the details of leg kinetic velocity, and the cross-terms lead to a degradation of identification accuracy according to the comparisons between WVD and SPWVD.

Furthermore, the trunk movement data are also important to identify personal gait, even though we can consider that these would not lead to differences in the results for various time-velocity distributions. Previous research on biomechanics has shown that trunk velocity and acceleration during walking contain essential information about individual gait, such as gait changes caused by the differences in physical and cognitive functions [38,39]. Therefore, trunk motion is essential for individual gait identification. However, approximately all of our generated time-velocity distribution images clearly include trunk motion, and this can be considered as the factor that indicates accurate personal identification to a certain extent for all time-velocity distributions and various resolution settings. However, this factor on the trunk movement does not lead to large differences in identification accuracy between the various time-velocity distribution images because of the clear trunk movement data.

In contrast, received power also reflects information about the location and shape of the trunk, legs, and arms, and this information includes personal information. However, as shown in Figure 2, although the trend of the distribution of received power is the same to some extent, the details of received power are not highly reproducible in the same person. Therefore, the factor of received power does not appear to be affected to a greater extent than trunk and leg movements.

Based on the above discussions, it can be considered that leg movement is an important factor in radar-based gait identification. In addition, trunk motion appears to be a factor contributing to the high accuracy of our results, although it does not appear to be significantly affected by the differences between the various types of time-velocity distribution images that we are primarily examining in this study.

### 4.6. Comparison with Other Studies

Our results were compared to those of conventional studies on Doppler-radar-based gait identification to further clarify the merits and novel findings in this study. Table 4 summarizes the main characteristics of the studies on gait-based personal identification by applying deep learning to Doppler radar data. The results of this study outperform various methods in terms of accuracy and shortness of the input data. However, because the experimental settings, including radar specifications and positions, are different for all studies, the accuracies listed in Table 4 should be treated as references. Nonetheless, our study suggests the effectiveness of resolution tuning for the time-velocity distribution in personal gait identification, as most conventional methods rely on empirically tuned STFT spectrograms with fixed time and velocity resolutions. That is, this study investigated the best window length, and the most important finding of this study was that a relatively shorter window length achieved better identification accuracy. The window length was significantly shorter than that in all other conventional studies with respect to its ratio to the length of the input data.

The results in [33] indicated similar accuracies for the STFT and SPWVD methods with respect to the effectiveness of various time-velocity distributions, because their resolution tunings were not considered. However, we noted that the identification accuracy of the optimally tuned SPWVD method was lower than that of the STFT method. Similar results were obtained using the WT and WVD methods. Thus, we demonstrated that an optimally tuned STFT is the most suitable method. These results implied that although WVD and SPWVD can identify personal gait with moderate accuracy, the cross-terms included in these distribution images may deteriorate the identification accuracy compared with the STFT.

Based on the novel results obtained from our investigations, the effectiveness of shorter input data and suitable accuracy for a relatively larger number of people were achieved, as indicated in Table 4. These findings are also important for practical applications. The above results include various novel findings for the radar-based personal gait identification method with respect to the efficient time-velocity distribution images and their resolutions. Thus, we compared the results obtained using the various time-velocity distributions to clarify the findings and contributions of this study.

## 5. Conclusions

To find the effective time-velocity distribution images for Doppler-radar-based personal gait identification using a CNN, four types of representative time-velocity analysis methods and their parameter settings were explored: STFT, WT, WVD, and SPWVD. Experimental investigations to identify 25 test subjects determined the appropriate parameters that controlled the time and velocity resolution for all methods. As a result, the highest accuracy of 99.1% was achieved with the optimally tuned STFT spectrogram images. The results revealed that the performance of the STFT method, which is generally used for radar-based gait identification, was superior to that of WT, WVD, and SPWVD in terms of high-resolution time-velocity distributions. A significant finding made during this study was that the high-resolution time-velocity distributions do not necessarily lead to highly accurate individual identification because of the cross-terms in WVD. We also revealed that the shorter window function for the STFT is effective for gait identification.

The primary limitation of this study is the small sample size, with 25 participants, 22 of whom are male. Therefore, our dataset may not be sufficient for practical application in many authentication scenarios. Therefore, future research should involve a larger number of participants and data, particularly data from female participants. Another significant limitation is that the study only considered participants walking towards the radar. Future research should demonstrate that the resolution tuning of the time-velocity distribution is also effective for gait identification using data from participants walking in arbitrary directions. Furthermore, the combination of time-velocity distributions generated via other methods was not considered. The achievement of more accurate identification by combining various types of time-velocity distributions is a promising direction for the advancement of biometric technology.

## Figures and Tables

**Table 1:** Results for STFT spectrogram.

	No. of Participants *N*		
Window Length *W*_L_	5	15	25
4 samples (6.67 ms)	88.0 ± 2.2%	76.0 ± 2.0%	70.4 ± 1.7%
8 samples (13.3 ms)	94.6 ± 1.8%	93.6 ± 1.1%	93.4 ± 0.7%
16 samples (26.7 ms)	98.4 ± 1.1%	98.2 ± 0.3%	98.8 ± 0.6%
**32 samples (53.3 ms)**	**98.8 ± 0.3%**	**99.3 ± 0.2%**	**99.1 ± 0.4%**
64 samples (0.107 s)	98.2 ± 0.9%	98.9 ± 0.4%	98.5 ± 0.4%
128 samples (0.213 s)	98.7 ± 0.8%	98.0 ± 0.5%	98.5 ± 0.2%
256 samples (0.427 s)	91.8 ± 1.4%	92.8 ± 1.3%	94.3 ± 0.6%

**Table 2:** Results for WT Scalogram.

		No. of Participants *N*		
*b*	*γ*	5	15	25
8	3	96.0 ± 1.3%	95.8 ± 0.6%	96.8 ± 0.7%
8	8	96.8 ± 1.1%	96.7 ± 0.9%	97.4 ± 0.7%
16	3	96.7 ± 1.0%	96.9 ± 0.7%	97.4 ± 0.4%
16	8	96.4 ± 0.3%	97.0 ± 0.7%	98.1 ± 0.4%
32	3	97.0 ± 0.9%	97.5 ± 0.8%	97.7 ± 0.4%
**32**	**8**	**97.9 ± 1.0%**	**97.8 ± 0.6%**	**98.1 ± 0.3%**

**Table 3:** Results for WVD and SPWVD.

			No. of Participants *N*		
	*α*	*β*	5	15	25
WVD	-	-	94.2 ± 1.5%	92.0 ± 1.1%	92.9 ± 1.0%
SPWVD	4	4	93.4 ± 1.6%	94.2 ± 1.2%	**95.2 ± 0.8%**
	4	64	**95.6 ± 1.0%**	**95.1 ± 0.9%**	95.0 ± 0.6%
	64	4	92.7 ± 2.5%	90.7 ± 1.3%	93.1 ± 0.4%
	64	64	94.5 ± 1.4%	92.2 ± 0.9%	92.1 ± 0.9%

**Table 4:** Comparison of Different Studies on Doppler-Radar-Based Personal Gait Identification.

Study	No. of Persons	Time-Velocity Distribution	STFT Window Length [s]	Length of Each Input Data Point [s]	Accuracy [%]
[17]	20	STFT	0.064	1	84.6
				3	96.7
[19]	4	STFT	0.2	1	96.8
[22]	4	STFT	0.13	2	97.1
	20				68.9
[23]	15	STFT	0.2	1	94.4
[33]	7	STFT	Not provided	4.5	86.4
		SPWVD	N/A	4.5	85.8
**This study**	**25**	**WT**	**N/A**	**Approx. 1 s** **(1 gait cycle)**	**98.1**
		**WVD**	**N/A**		**92.9**
		**SPWVD**	**N/A**		**95.2**
		**STFT**	**0.013**		**93.4**
			**0.053**		**99.1**
			**0.43**		**94.3**

## Data Availability

The data are available upon reasonable request.
